# Impact of Human Mobility on COVID-19 Transmission According to Mobility Distance, Location, and Demographic Factors in the Greater Bay Area of China: Population-Based Study

**DOI:** 10.2196/39588

**Published:** 2023-04-26

**Authors:** Jizhe Xia, Kun Yin, Yang Yue, Qingquan Li, Xiling Wang, Dongsheng Hu, Xiong Wang, Zhanwei Du, Ben J Cowling, Erzhen Chen, Ying Zhou

**Affiliations:** 1 Department of Urban Informatics Guangdong Key Laboratory for Urban Informatics Shenzhen University Shenzhen China; 2 Guangdong Laboratory of Artificial Intelligence and Digital Economy Shenzhen China; 3 School of Global Health Chinese Center for Tropical Diseases Research Shanghai Jiao Tong University School of Medicine Shanghai China; 4 School of Public Health and Key Laboratory of Public Health Safety Fudan University Shanghai China; 5 School of Public health Shenzhen University Shenzhen China; 6 Institute for Advanced Study Shenzhen University Shenzhen China; 7 World Health Organisation Collaborating Centre for Infectious Disease Epidemiology and Control School of Public Health, Li Ka Shing Faculty of Medicine The University of Hong Kong, Hong Kong Special Administrative Region Hong Kong China (Hong Kong); 8 Laboratory of Data Discovery for Health Hong Kong Science and Technology Park Hong Kong China (Hong Kong); 9 Ruijin Hospital Shanghai Jiao Tong University School of Medicine Shanghai China

**Keywords:** COVID-19, mobility restriction, mobility distance, demographic factors, locations

## Abstract

**Background:**

Mobility restriction was one of the primary measures used to restrain the spread of COVID-19 globally. Governments implemented and relaxed various mobility restriction measures in the absence of evidence for almost 3 years, which caused severe adverse outcomes in terms of health, society, and economy.

**Objective:**

This study aimed to quantify the impact of mobility reduction on COVID-19 transmission according to mobility distance, location, and demographic factors in order to identify hotspots of transmission and guide public health policies.

**Methods:**

Large volumes of anonymized aggregated mobile phone position data between January 1 and February 24, 2020, were collected for 9 megacities in the Greater Bay Area, China. A generalized linear model (GLM) was established to test the association between mobility volume (number of trips) and COVID-19 transmission. Subgroup analysis was also performed for sex, age, travel location, and travel distance. Statistical interaction terms were included in a variety of models that express different relations between involved variables.

**Results:**

The GLM analysis demonstrated a significant association between the COVID-19 growth rate ratio (GR) and mobility volume. A stratification analysis revealed a higher effect of mobility volume on the COVID-19 GR among people aged 50-59 years (GR decrease of 13.17% per 10% reduction in mobility volume; *P*<.001) than among other age groups (GR decreases of 7.80%, 10.43%, 7.48%, 8.01%, and 10.43% for those aged ≤18, 19-29, 30-39, 40-49, and ≥60 years, respectively; *P*=.02 for the interaction). The impact of mobility reduction on COVID-19 transmission was higher for transit stations and shopping areas (instantaneous reproduction number [R_t_] decreases of 0.67 and 0.53 per 10% reduction in mobility volume, respectively) than for workplaces, schools, recreation areas, and other locations (R_t_ decreases of 0.30, 0.37, 0.44, and 0.32, respectively; *P*=.02 for the interaction). The association between mobility volume reduction and COVID-19 transmission was lower with decreasing mobility distance as there was a significant interaction between mobility volume and mobility distance with regard to R_t_ (*P*<.001 for the interaction). Specifically, the percentage decreases in R_t_ per 10% reduction in mobility volume were 11.97% when mobility distance increased by 10% (Spring Festival), 6.74% when mobility distance remained unchanged, and 1.52% when mobility distance declined by 10%.

**Conclusions:**

The association between mobility reduction and COVID-19 transmission significantly varied according to mobility distance, location, and age. The substantially higher impact of mobility volume on COVID-19 transmission for longer travel distance, certain age groups, and specific travel locations highlights the potential to optimize the effectiveness of mobility restriction strategies. The results from our study demonstrate the power of having a mobility network using mobile phone data for surveillance that can monitor movement at a detailed level to measure the potential impacts of future pandemics.

## Introduction

### Background

The COVID-19 pandemic had led to over 630 million infections and 6 million deaths worldwide by November 2022 [1]. Human mobility is critical to the transmission of airborne diseases. Reduction in human mobility was considered as one of the main tools to suppress and mitigate the pandemic, and has been applied widely and even repeatedly in multiple waves globally [2]. However, frequent mobility restrictions globally have caused unprecedented public health, social, and economic challenges. For example, mobility reduction was associated with indirect health outcomes, such as increased cerebrovascular excess deaths [3] and increased mental health issues [4]. Substantial economic and social burdens of lockdown measures were reflected in regions with different financial capacities [5]. The vaccine program may fail to help reach herd immunity, and easing mobility restrictions should be carefully considered against the risk of new outbreaks [6]. When variants with higher transmissibility circulate globally, mobility restriction could be one of the primary tools to control the pandemic. Therefore, it is important to determine how to optimize such mobility restriction measures to balance the indirect adverse outcomes in the society and economy, and the benefits of controlling transmission risk for COVID-19.

In almost 3 years of the COVID-19 pandemic, unprecedented efforts have been made to explore the association between human mobility and COVID-19 transmission [7]. Mobile-based mobility data can be used to assess mobility reduction and the effectiveness of social distancing for mitigating disease spread [8,9]. Most studies used mobility volume (ie, number of trips) for investigating the impact of mobility restriction policies on the transmission of COVID-19, but the correlation between reductions in mobility volume and COVID-19 transmission became weaker and even disappeared in the middle stage of the pandemic according to 2 studies in the United States [10,11]. Another study using the data of 52 countries indicated that the association between mobility volume and the reproduction number R changed over time [12]. After public health interventions were relaxed, the association between mobility and COVID-19 transmission decoupled in most countries. More detailed research is needed to better understand such a relation. Very few studies have explored the association between mobility distance and COVID-19 transmission [13], and it is unclear whether the weaker impact of mobility volume was influenced by a disproportional change in mobility distance. COVID-19 is known to exhibit age-related severity, and exposure rates are strongly dependent on age [14]. Similar mobility reductions among <18, 18-64, and ≥65 years age groups were reported in France [15], but it is unclear whether such reductions in various age groups have similar effects on COVID-19 transmission. Governments around the world have different policies for implementing and easing social distancing measures in various locations in the absence of evidence. These locations may have different transmission risks and social benefits [16]. It is critical to differentiate the impact of mobility reduction for various locations when making decisions to shut down specific locations. The impact of mobility reduction on COVID-19 transmission according to travel distance, location, and demographic factors is of great importance for understanding mobility restriction policies, but it has been poorly explored.

### Objectives

In this study, we integrated anonymized geolocalized mobile phone data with census and demographic data in the Greater Bay Area of China. We aimed to analyze the impact of mobility reduction on COVID-19 transmission according to travel distance (long and short), location (workplaces, schools, recreation areas, shopping areas, transit stations, and other areas), age (≤18, 19-29, 30-39, 40-49, 50-59, and ≥60 years), and sex. The coverage rate of mobile phone use among the population aged 15-65 years was almost 100% [17]. This analysis could provide evidence to optimize mobility restriction policies for balancing the adverse outcomes of mobility reduction and the benefits of limiting community transmission of COVID-19.

## Methods

### Data Source

#### Mobility Data From the Greater Bay Area, China

Large volumes of anonymized aggregated mobile phone position data between January 1 and February 24, 2020, were collected for the 9 megacities of Guangzhou, Shenzhen, Foshan, Huizhou, Dongguan, Zhongshan, Zhaoqing, Zhuhai, and Jiangmen in Guangdong-Hong Kong-Macao Greater Bay Area, China. The Greater Bay Area is the most populated and largest urban area, and is 1 of the 4 largest bay areas in the world. Mobile phone data were provided by 1 of the 3 leading mobile phone service providers. Origin-destination matrices were constructed by computing the number of people that move between different locations on an hourly basis, as done previously [18]. The mobility volume was calculated as the number of trips between various locations. The mobility distance was determined by calculating the great circle distance between movement origins and destinations [19]. Aggregated sex- and age-specific daily mobility volumes were also obtained. Daily mobility volumes to various destinations (eg, workplaces, schools, recreation areas, shopping areas, transit stations, and other areas) were calculated by integrating the origin-destination matrices with the land use type of the trip destination (Table S1 in Multimedia Appendix 1). Official estimates of the total, sex-specific, and age-specific populations in the Greater Bay Area were retrieved from relevant government websites [20].

#### COVID-19 Transmission Data

Daily incidences of COVID-19 were obtained from official governmental reports in the Greater Bay Area, China [21]. Data on country-level estimates of the instantaneous reproduction number (R_t_) were obtained from the EpiForecasts project by the London School of Hygiene & Tropical Medicine (London, UK) [22], which was calculated based on the daily number of COVID-19 infections by the European Centre for Disease Prevention and Control. The timings and details for public health interventions were taken as published on relevant government websites in the Greater Bay Area, China [23].

### Ethics Approval

We used anonymized and aggregated mobile phone data at the population level without individual travel patterns for strict protection of personal privacy. All data were obtained in an anonymous format without personal identifying information. This study was approved by the Institutional Review Board of Shenzhen University, China (review number PN-202300030).

### Statistical Analysis

The growth rate ratio (GR) of COVID-19 was computed as the average number of new cases per day over the previous 3 days to that over the previous 7 days. The static correlation and dynamic correlation between the GR and mobility volume were determined by Pearson correlation and rolling correlation. We tested the different day lags between the GR and mobility in the correlation analysis, as there may be a time lag between reported cases and true community infections. A generalized linear model (GLM) was established to test the association between mobility volume and COVID-19 transmission. Subgroup analysis was also performed for sex, age, travel location, and travel distance. The statistical interaction terms were included in a variety of models that express different relations between the involved variables. The R_t_ in Shenzhen by time for real transmission was estimated according to the likelihood-based estimation method [24].

An interaction analysis was performed by including the interaction terms of mobility volume ratio (VR) and distance ratio (DR) in the GLM analysis to determine whether the impact of mobility volume on COVID-19 transmission differs by mobility distance in the Greater Bay Area, China. The VR and DR were defined for each day (t), which quantified the change in mobility patterns and were similar to previous studies [25]. The baseline dates for the VR and DR were the normal days in 2020, which are here defined as the days before *Chunyun* (Spring Festival; January 1-10, 2020; Multimedia Appendix 1) for the Greater Bay Area, China. The VR is the sum of the total trips between various locations on a given day divided by the same measure on the baseline day, which reflects the change in the number of individual trips made to each area per day. *VR_it_* was calculated as follows:



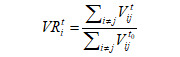



where *V_ij_^t^* represents the number of trips between areas *i* and *j* on day *t*, and *t_0_* represents the baseline measure. Using this function, VR values of 0, 0.5, and 1.0 indicate no trips, half the number of trips relative to baseline, and no change compared with baseline, respectively.

The DR represents the change in the distance of individual trips made to each area per day, relative to ordinary behavioral patterns (ie, before COVID-19), which was calculated as follows:



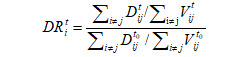



where *D_ij_^t^* represents the distance of trips between areas *i* and *j* on day *t*, and *t_0_* represents the baseline measure. DR values of 0, 0.5, and 1.0 indicate no trips, half of the mobility distance at baseline, and no change compared with baseline, respectively. Any value above 1 indicates that the mobility distance increased from baseline. The R_t_ ratio was defined as the R_t_ on a given day compared to the first R_t_ at the beginning of the study. ANOVA was used to evaluate the model with and without the interaction term. All statistical analyses were performed using R version 3.6.3 (R Foundation for Statistical Computing).

## Results

### The Effect of Mobility Volume on COVID-19 Transmission

**Figure 1:**
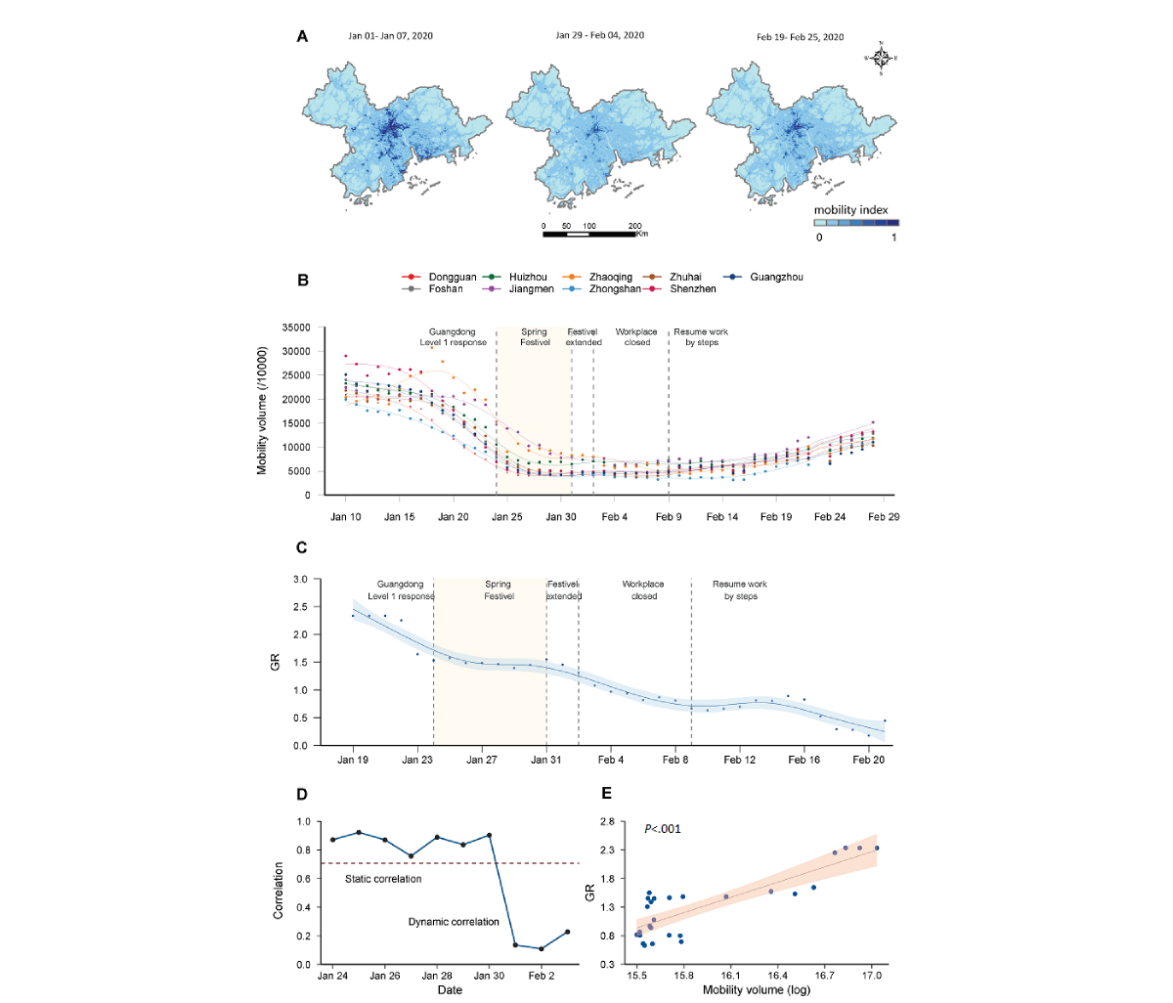
Relationship between mobility volume and the GR in the Greater Bay Area, China. (A) Average mobility volume based on the mobile phone position data from January to February 2020 in the Greater Bay Area, China. (B) The change in mobility volume for 9 cities from January to February 2020 (the dots represent the observed data, and the plotted lines are smoothed by a generalized additive model). (C) The GR of COVID-19 from January to February 2020 (the line represents GLM fit to the data, and the shadow represents the 95% CI). (D) Correlation between mobility volume and the GR. (E) Association between mobility volume and the GR (the line represents GLM fit to the data, and the shadow represents the 95% CI). The dashed lines represent the main public health interventions. GLM: generalized linear model; GR: growth rate ratio.

The public health interventions in the Greater Bay Area, China during the study period are illustrated in Multimedia Appendix 2. The population mobility volume decreased by approximately 75%-85% and then was maintained at such a substantially low level for approximately 2 weeks in January 2020 in the Greater Bay Area, China (Figure 1A and 1B). The GR gradually decreased from 2.33 on January 19, 2020, to below 0.50 on February 20, 2020 (Figure 1C). The static correlation between the GR and mobility volume was 0.71 (*P*<.001; Figure 1D), considering the time lag of 2 days representing the highest correlation between the GR and mobility volume (Multimedia Appendix 3). The rolling correlation coefficients were >0.75 from January 24, 2020, to January 31, 2020 (all *P*<.01) but declined in February 2020. The GLM analysis demonstrated a significant association between the GR and mobility volume, with a 7.56% decrease in the GR per 10% reduction in mobility volume (95% CI 6.13%-9.00%; *P*<.001; Figure 1E).

### Demographic Disparities in the Impact of a Reduction in Mobility Volume on COVID-19 Transmission

**Figure 2:**
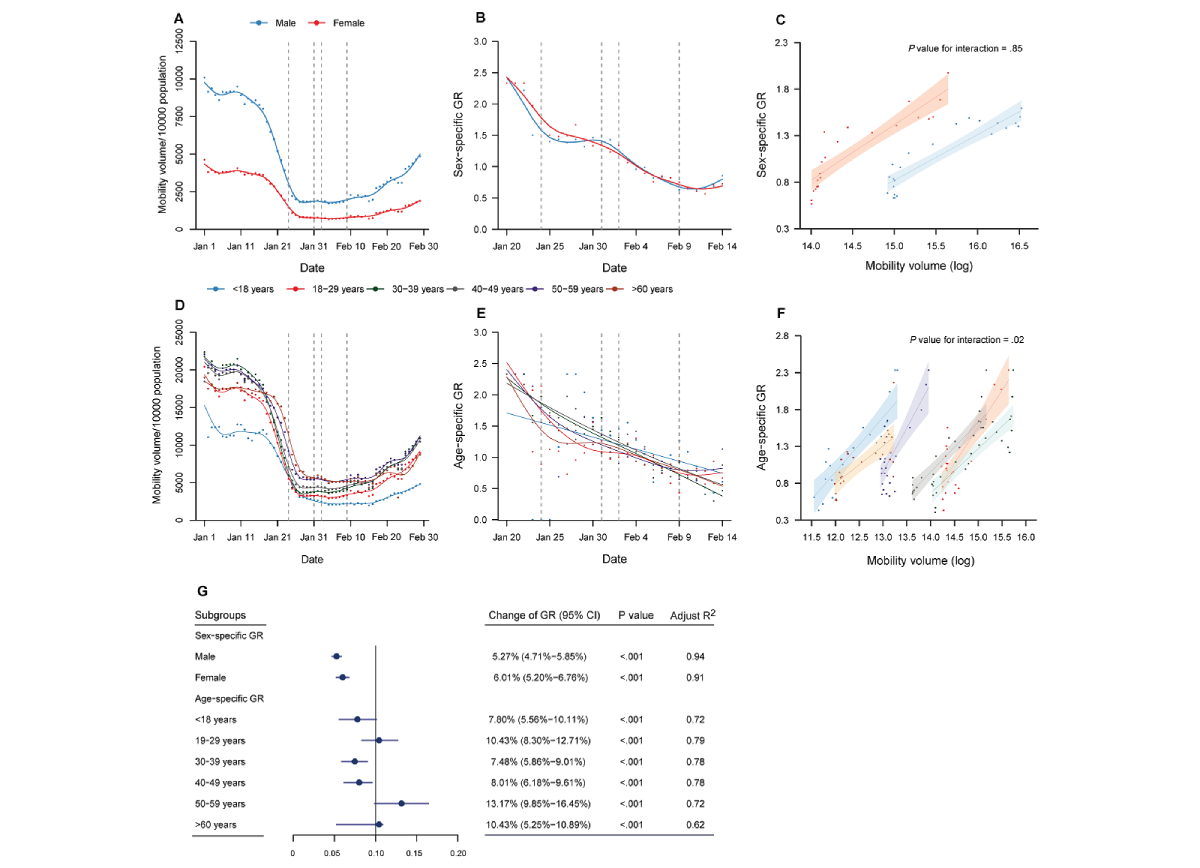
Relationship between mobility volume and the GR by sex and age. (A) Time series of the daily average mobility volume and (B) the sex-specific GR for males and females. The dots represent the raw data, while the plotted lines are smoothed by a generalized additive model. (C) Relationship between the sex-specific GR and sex-specific mobility volume. The line represents GLM fit to the data, and the shadow represents the 95% CI. (D) Time series of the daily average mobility volume and (E) the age-specific GR for various age groups. The dots represent the raw data, while the plotted lines are smoothed by a generalized additive model. (F) Relationship between the age-specific GR and age-specific mobility volume. The line represents GLM fit to the data, and the shadow represents the 95% CI. The dashed lines represent the main public health interventions. (G) The change in the demographic-specific GR per 10% reduction in mobility volume. GLM: generalized linear model; GR: growth rate ratio.

We next investigated the impact of a reduction in mobility volume on COVID-19 transmission in various subgroups of sex and age. Although the mobility volume for females was less than that for males before, during, and after the COVID-19 pandemic (Figure 2A), a similar magnitude of decrease was observed (77.4% for females and 77.8% for males during the pandemic; Multimedia Appendix 4). The sex-specific GR declined with similar trends for both sexes (Figure 2B). The slopes of the association between the sex-specific mobility volume and sex-specific GR (Figure 2C and 2G) were similar in the GLM analysis for females and males.

Among different age groups, those aged 50-59 and ≥60 years exhibited the lowest levels of reduction in mobility volume of 72.9% and 67.0%, respectively, during the pandemic. The percentage reductions in mobility volume were 78.1%, 81.5%, 81.4%, and 77.7% for those aged ≤18, 19-29, 30-39, and 40-49 years, respectively (Figure 2D; Multimedia Appendix 4). The slopes of the association between the age-specific mobility volume and age-specific GR were significantly different among various age groups. The percentage decreases in the GR per 10% reduction in mobility volume were 7.80% for those aged ≤18 years (*P*<.001), 10.43% for those aged 19-29 years (*P*<.001), 7.48% for those aged 30-39 years (*P*<.001), 8.01% for those aged 40-49 years (*P*<.001), 13.17% for those aged 50-59 years (*P*<.001), and 10.43% for those aged ≥60 years (*P*<.001) (*P*=.02 for the interaction) (Figure 2F and 2G). The age group of 50-59 years had the highest slope in the GLM analysis for the age-specific GR and age-specific mobility volume.

### Location Disparities in the Impact of a Reduction in Mobility Volume on COVID-19 Transmission

**Figure 3:**
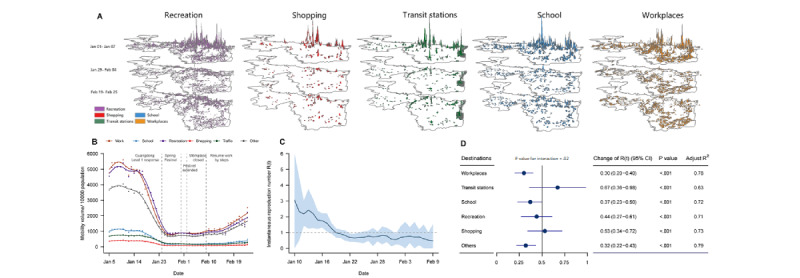
Relationship between mobility volume and COVID-19 transmission by various destinations. (A) The distribution of mobility volume for various destinations in the city of Shenzhen at different time periods in the Greater Bay Area, China (the peak of 3D bars represents the mobility volume in the given period), and (B) the time series of mobility volume at these destinations. (C) Time series of the instantaneous reproduction number (Rt) in Shenzhen. The shadow represents the 95% CI. (D) The change in the Rt per 10% reduction in mobility volume for a certain destination. Adjusted R^2^ represents the goodness of fit for the models.

The distribution of mobility volume at 6 types of locations at different time periods in the city of Shenzhen (one of the Greater Bay Area cities) is illustrated in Figure 3A. The mobility volume for workplaces was notably higher than other locations. The mobility volume at the 6 types of locations had declined during the pandemic, with average reductions of 83.8% for workplaces, 75.1% for transit stations, 83.5% for shopping areas, 82.9% for recreation areas, 83.7% for schools, and 81.8% for other locations (Figure 3B). The impact of mobility volume on the R_t_ was higher for the locations of transit stations and shopping areas, with the highest slope in the GLM analysis. The decreases in the R_t_ per 10% reduction in mobility volume were 0.67 for transit stations (*P*<.001), 0.53 for shopping areas (*P*<.001), 0.30 for workplaces (*P*<.001), 0.37 for schools (*P*<.001), 0.44 for recreation areas (*P*<.001), and 0.32 for other locations (*P*<.001) (*P*=.02 for the interaction; Figure 3D).

### Mobility Distance Disparities in the Impact of a Reduction in Mobility Volume on COVID-19 Transmission

**Figure 4:**
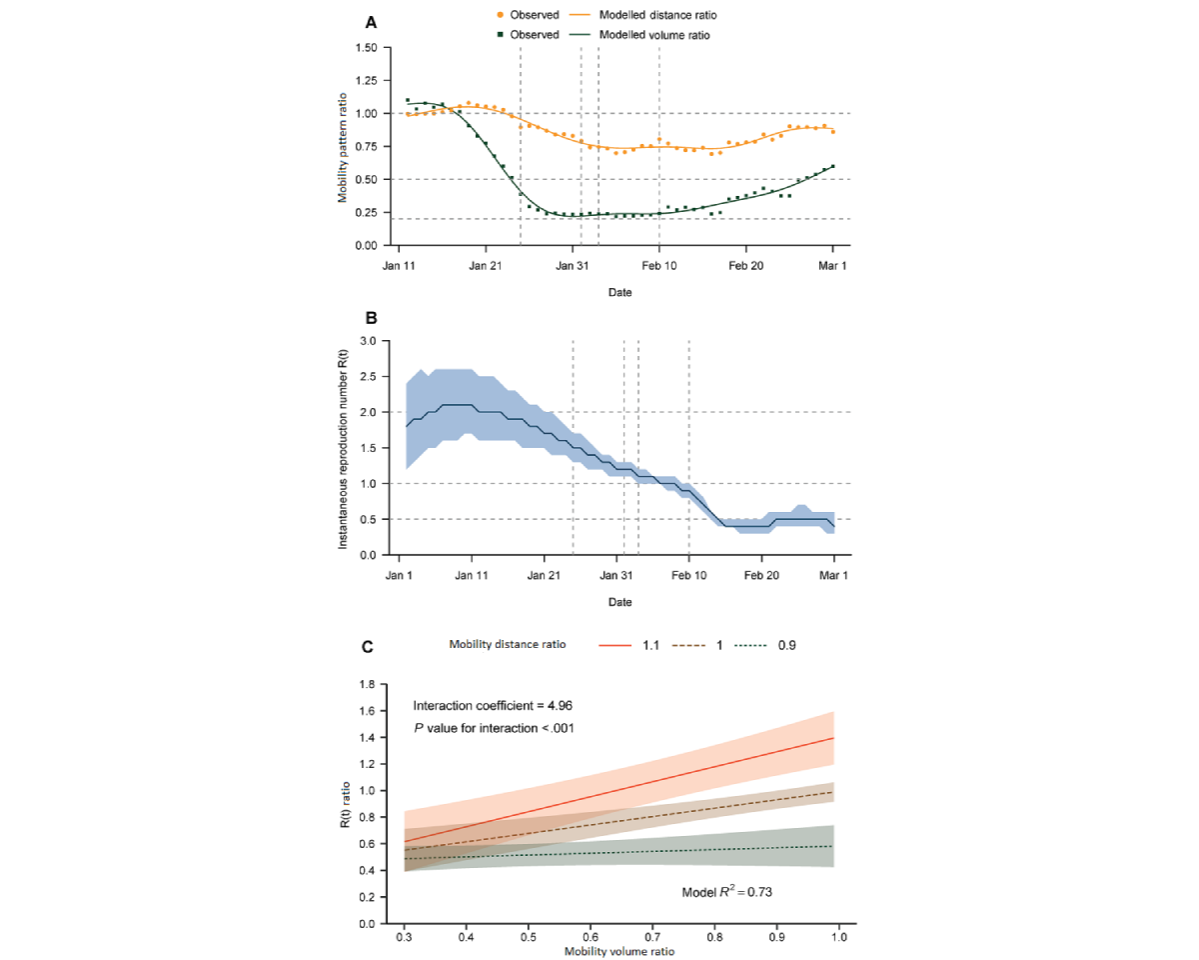
Relationship between mobility volume and COVID-19 transmission by mobility distance. (A) Time series of the VR and DR in the Greater Bay Area, China. The dots represent the observed data, and the plotted lines are smoothed by a generalized additive model. (B) Time series of the instantaneous reproduction number (Rt) in the Greater Bay Area, China. The shadow represents the 95% CI. (C) Relationship between the VR and Rt ratio by different DRs in the Greater Bay Area, China. The line represents GLM fit to the data, and the shadow represents the 95% CI. DR: distance ratio; GLM: generalized linear model; VR: volume ratio.

The change in mobility volume occurred earlier and was greater than the change in distance during the pandemic in the Greater Bay Area, China (Figure 4A). The population mobility volume had an average reduction of 76.1% in February compared with the baseline period of 2020 in the Greater Bay Area, China, and the mobility distance declined by an average of 18.6% (Figure 4A). The R_t_ had reduced to <1 by February 8, 2020, in China (Figure 4B). Exploratory analyses showed statistical evidence of an interaction between the VR and DR for the COVID-19 R_t_ ratio. In the analysis of Chinese cities, the impact of a reduction in mobility volume on the R_t_ was lower with a decreasing mobility distance. The percentage decreases in the R_t_ per 10% reduction in mobility volume were 11.97% when the average mobility distance increased by 10% compared with the baseline period, such as in the *Chunyun* period when people travelled longer distances in the Spring Festival (*P*<.001), 6.74% when the average mobility distance remained unchanged (*P*<.001), and 1.52% when the distance declined by 10% (*P*<.001) (*P*<.001 for the interaction; Figure 4C).

ANOVA showed that removing the interaction did significantly affect the fit of the model (*P*<.001). The R^2^ for the model without an interaction was 0.65, which was lower than that for the model with an interaction (0.73 in the China model), increasing the variance of the dependent variable explained by the predictors. Finally, we validated the mobile phone mobility data for the Greater Bay Area, China by comparing it to Baidu mobility data for the whole country [26]. Here, the trend of mobility volume reduction was quite similar to the change in the mobility index from the Baidu Qianxi map (Multimedia Appendix 5).

## Discussion

### Principal Findings

In this study, we found that the impact of reductions in human mobility on COVID-19 transmission significantly varied by travel distance, location, and age. There was a significant positive interaction between mobility volume and mobility distance regarding COVID-19 transmission, with steeper slopes for the association (larger coefficient in the regression analysis) between mobility volume and COVID-19 transmission with increasing mobility distance. We found a significantly steeper slope for the association between the reduction in mobility volume and COVID-19 transmission among persons aged 50-59 years than among other age groups. Furthermore, the slope for the association was steeper for the locations of transit stations and shopping areas, compared with workplaces, schools, recreation areas, and other locations.

Our study indicated that the introduction of mobility restrictions in the Greater Bay Area, China led to a marked decrease in COVID-19 transmissibility. The time lag between mobility reduction and decline in the GR was estimated as 2 days at the very beginning of the pandemic in this area, which is shorter than the time of around 2 weeks in a similar study in the United States reported by Badr et al [11]. Our study also showed that the R_t_ in Shenzhen City, one of the megacities in this area, had declined from >3 to <1 within around 2 weeks, which means that the public health interventions in this area worked very fast. Therefore, the shorter time lag of 2 days between mobility reduction and change in the GR may be possible in the Greater Bay Area, China. The reduction in mobility volume with longer travel distances was associated with a greater reduction in COVID-19 transmission than shorter travel distances in our study. The decreasing correlations of mobility volume with COVID-19 transmission observed in our study were consistent with previous studies in the United States. Badr et al [11] found a strong correlation between mobility volume and COVID-19 case growth rates in the early stage of March to April 2020, but a strong linear association was absent after April 2020. Gatalo et al [10] also identified a strong correlation between March 27, 2020, and April 20, 2020, and only a weak correlation at later time periods (April 21, 2020, to May 24, 2020). The significant positive interaction between mobility volume and distance may provide a possible explanation for the time-driven relationship between mobility volume and COVID-19 transmission. The changes in mobility distance and volume are not necessarily synchronized. Our study indicated that there is an interaction, and changes in moving distance may affect the impact of mobility volume on COVID-19 transmission. This provides evidence for stricter restrictions in long-distance travel for better control of COVID-19 transmission. However, the different start times and levels of reduction in mobility volume and distance during the pandemic need to be further explored.

The mobility reduction was associated with a greater reduction in the GR for the age group of 50-59 years than the other age groups in our study. The slope of the association between mobility volume and the GR for those aged 50-59 years was steeper than that for the other age groups. Those aged 50-59 years showed lower mobility reduction than those aged ≤49 years, but were vulnerable to COVID-19 infection with a higher proportion of underlying chronic diseases compared with young people [27]. Those aged 50-59 years faced a higher risk of exposure to SARS-CoV-2 with more traveling for occupational and behavioral reasons compared with those aged ≥60 years [28]. These factors may provide possible explanations for the finding that most infections of COVID-19 in the initial wave in China were among those aged 50-59 years (22.4%) [29]. The highest proportion of COVID-19 cases was also among those aged 50-59 years during the initial wave from January to May 2020, but shifted toward younger people from June to August 2020 in the United States [30]. A similar age shift was observed in Europe, with the median age of patients with COVID-19 declining from 54 years at the beginning to 39 years in the later time periods [31]. Whether such an age shift is associated with a change in the mobility pattern for various age groups at the different stages of the pandemic needs further exploration. In addition, our data suggested that the declined magnitude of mobility volume and the effect of mobility volume on COVID-19 transmission were quite similar for both males and females, which is consistent with the conclusion of no sex variation for COVID-19 infections in China [32].

The mobility reduction for transit stations and shopping centers was associated with a greater reduction in COVID-19 transmission in the whole city compared with the findings for workplaces, schools, and recreation areas. The slopes for the association between mobility to transit stations and shopping centers and the R_t_ were steeper than the slopes for the association involving other locations. These high-contact environments are more crowded and therefore have a higher risk. However, the mobility reduction for transit stations was less than that for other locations. Many governments applied various policies for mobility restriction at specific locations, since there was not enough evidence available regarding which locations should be closed or which locations should remain open. Our study provides evidence that controlling mobility to a small number of locations could reduce transmission in the entire city. Therefore, location-specific mobility restrictions should be taken into consideration for precise interventions and reopening strategies with substantially lower economic costs [33].

### Strengths and Limitations

Our results have public health and policy implications. First, we analyzed the relationship between mobility responses and COVID-19 transmission using mobility data of only a certain travel destination, travel distance, or demographic subgroup (age and sex groups) to gain more insightful knowledge. Our study provides evidence to identify hotspots of transmission and guide policy interventions for specific age groups or mobility patterns associated with higher risks of mobility-related COVID-19 transmission. Second, mobile phone data at fine spatial and temporal resolutions provide strong added value for explaining variations in COVID-19 transmission. The results from our study demonstrate the power of having mobility networks using mobile phone data that monitor movement at a detailed level across cities to measure the potential impacts of public health events. The network can be regularly updated and used to identify populations and travel characteristics at risk of adverse impacts during future pandemics or other crises. It is also suggested to set up a national mobility network that captures human mobility habits, which can form the basis for longitudinal studies.

It is important to note that our study has several limitations. First, we illustrated the detailed structural change in mobility patterns using mobile phone data from users in the Greater Bay Area, China, and these patterns may not be fully representative of other locations in China. However, the mobility change in the Greater Bay Area based on the mobile phone was quite similar to the change in the Baidu mobility index for the whole country, owing to nationally unified public health interventions. We believe that this analysis for the Greater Bay Area is an intuitive and representative estimate of the structural change in mobility patterns in China, but future extension of this analysis for the whole country should be further explored. Second, we focused on quantifying the relationship between mobility patterns and COVID-19 transmission; there is extensive evidence indicating that population-wide social distancing and other potential mitigating factors (eg, wearing face masks and washing hands) all contribute to achieving control of the COVID-19 pandemic [34,35]. Further studies are needed to evaluate the synergistic effect of other public health interventions along with mobility change in the environment confounded by climate, urban space, and other potential factors [36]. Third, our mobility estimates may also be biased toward the populations included in the mobile phone data, as the consumer location history feature is only available for smartphone users (young infants or very old people may be excluded from the data). However, despite these limitations, our fine-grained study facilitates a step toward using multiple data sets to capture population-level mobility patterns and provides important insights into the complex effects of mobility reduction for policymakers and future research.

### Conclusions

The COVID-19 pandemic was the first time in human history that human mobility showed a large-scale decline after mobility restrictions to prevent and control the infectious disease. Our study demonstrated that the impact of reductions in human mobility on COVID-19 transmission was significantly modified by travel distance, travel location, and age. The higher impact of mobility reduction on COVID-19 transmission for longer distances, certain age groups, and specific locations highlights the potential to optimize mobility restriction policies to balance adverse health, society, and economic outcomes and the benefits of controlling the spread of COVID-19. It is of great significance to understand the impact of mobility reduction on the spread of infectious diseases in detail, and it provides evidence for the prevention and control of future pandemics.

## Appendix

Multimedia Appendix 1 Supplementary materials and methods.

Multimedia Appendix 2 Main public health interventions in the Greater Bay Area, China during the study period.

Multimedia Appendix 3 Correlations between mobility volume and growth rate ratio at different time lags (in days).

Multimedia Appendix 4 The magnitude of mobility volume change for various demographic groups.

Multimedia Appendix 5 The time series of mobility volume from mobile phone data in the Greater Bay Area, China and the mobility index from Baidu for the whole country of China.

## Abbreviations

DR: distance ratio
GLM: generalized linear model
GR: growth rate ratio
Rt: instantaneous reproduction number
VR: volume ratio

## Data Availability

We purchased the mobile phone data (January to March 2020) from a service provider (China Unicom). Our data purchase agreement with China Unicom prohibits us from sharing the data with third parties, but interested parties can contact China Unicom to obtain the same data. We collated epidemiological data from publicly available data sources (government websites). All epidemiological information that was used has been documented in the article.
