# Supplementary material for: Impact of Human Mobility on COVID-19 Transmission According to Mobility Distance, Location, and Demographic Factors in the Greater Bay Area of China: Population-Based Study
Source: JMIR Public Health Surveill. 2023 Apr 26;9:e39588. doi: 10.2196/39588 (PMC10138924; doi:10.2196/39588)
Supplement: Multimedia Appendix 1 [file publichealth_v9i1e39588_app1.doc]

**Multimedia Appendix 1.** Supplementary materials and methods.

*Mobility data in GBA, China*

The GBA has a total area of 56,000 square kilometers and a total population of 70 million. Different trip purposes are inferred based on the land use type of trip destination (Table S1). For example, if a trip ends at a high school, this trip will be defined as an “education trip”.

**Table S1. Land-use type of National destination**

| **Destination** | **Destination Description** |
| --- | --- |
| Workplaces | Buildings for workplaces including commercial office building, government office building, and factory. |
| Recreation | Open space for recreational use  including parks, public beaches, tourist attractions, etc. |
| Transit stations | Public transport hubs such as subway, bus, airport, and train stations. |
| Education | Buildings and space for educational use including primary schools, middle schools, colleges, and universities. |
| Shopping | Buildings and space for shopping center, grocery markets, food warehouses, farmers, markets. |

The gender and age are gathered by the mobile-phone service provider during the mobile number application process. Different from many other countries, every Chinese mobile phone number is strictly linked to the user's photo ID (identification card or passport), which is requested by local laws. People will need to upload their photo ID when they apply for a mobile phone number. As a result, every mobile phone number is linked to the user's gender and user birthday, while other socio-demographic information (e.g. income and education) is not linked because it is not included in the Photo ID.

On the other hand, individual mobile-phone user information is strictly protected by the Chinese user privacy regulations. Therefore, we cannot access individual-level information from the mobile-phone service provider. To analyze human mobility, our mobile-phone service providers aggregated all travel information into 500x500 meter grids with a 30-minute temporal resolution (Figure S1). With this process, human mobility information is anonymously aggregated, and we can analyze population-level mobility without violating local regulations.


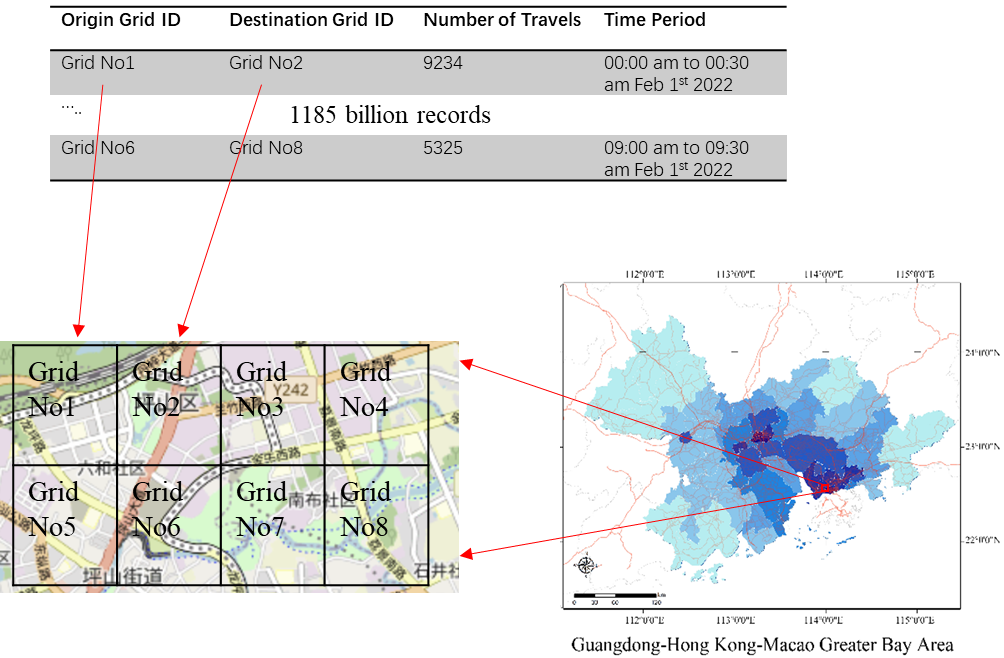


Figure S1 The data format of aggregated mobility data

The same as individual level user mobility information, individual level socio-demographic information (gender and age) is also strictly inaccessible to the public including research usage. Therefore, socio-demographic information is also aggregated in both space and time. Figure S2 provides an example of data aggregation formats. The original mobility data record is divided into two sub-datasets based on the socio-demographic classification. From the research side, no individual-level socio-demographic information is accessed but we can still analyze population-level mobility changes of different genders. A similar data aggregation process was conducted for the age group information.


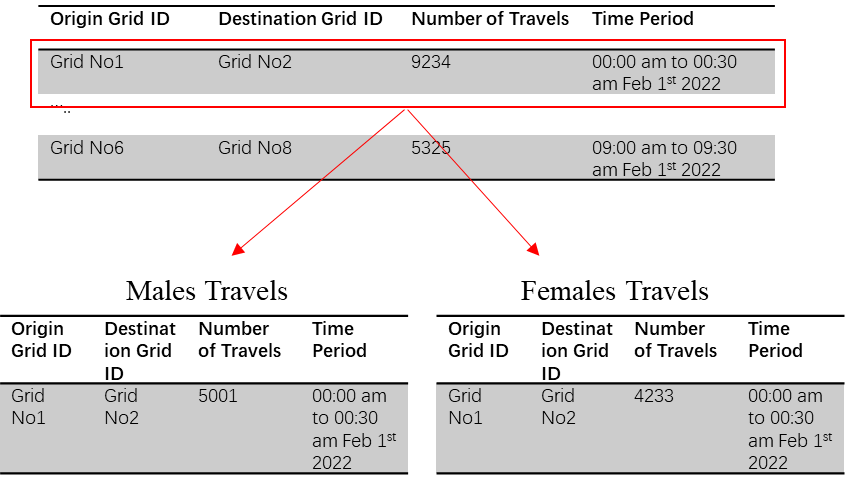


Figure S2 Aggregated mobility dataset with socio-demographic information

*Definition of Chunyun*

Chunyun is a period of the Spring Festival travel season before and after the Lunar New Year in China.The mobility during this period could remarkably change. People usually go back to less developed areas from developed areas such as cities in GBA during the 15 days before the Lunar New Year’s Day, and return to the developed areas after the Spring Festival. The period of *Chunyun* 2020 is from 10 January 2020 to 18 February 2020.

**Statistical analysis**

A generalized linear model (GLM) was established to test the association between mobility volume and COVID-19 transmission as the following:

glm (formula= log(mobility volume) ~GR)
