# Supplementary material for: Impact of Human Mobility on COVID-19 Transmission According to Mobility Distance, Location, and Demographic Factors in the Greater Bay Area of China: Population-Based Study
Source: JMIR Public Health Surveill. 2023 Apr 26;9:e39588. doi: 10.2196/39588 (PMC10138924; doi:10.2196/39588)
Supplement: Multimedia Appendix 2 [file publichealth_v9i1e39588_app2.doc]

**Multimedia Appendix 2.** Main public health interventions in the Greater Bay Area, China during the study period.

| **Date** | **Major Responses in the study period of 2020** |
| --- | --- |
| Jan.10-Jan.24 | - Start the joint prevention and control mechanism of major infectious diseases. - Close workplaces and schools and stop gathering activities. Citizens wear masks outside and enter public places with temperature screening. - Guangdong Province activated First-Level Public Health Emergency Response. |
| Jan.24-Jan.31 | - Extended the spring holiday in 2020. - Delayed school opening. - Closed community management. |
| Jan.31-Feb.2 | - Release the enterprise resumption of production reporting system. |
| Feb.3-Feb.9 | - The community continues to implement closed management, and residential building units with confirmed cases are isolated. |
| Feb.9-Feb.23 | - Orderly resumption of work and production after the Spring Festival holiday. |
| Feb.24-May.9 | - Guangdong Province lowered the risk level, from First-Level response to Two-Level response. |
