# Supplementary material for: Impact of Human Mobility on COVID-19 Transmission According to Mobility Distance, Location, and Demographic Factors in the Greater Bay Area of China: Population-Based Study
Source: JMIR Public Health Surveill. 2023 Apr 26;9:e39588. doi: 10.2196/39588 (PMC10138924; doi:10.2196/39588)
Supplement: Multimedia Appendix 3 [file publichealth_v9i1e39588_app3.doc]

**
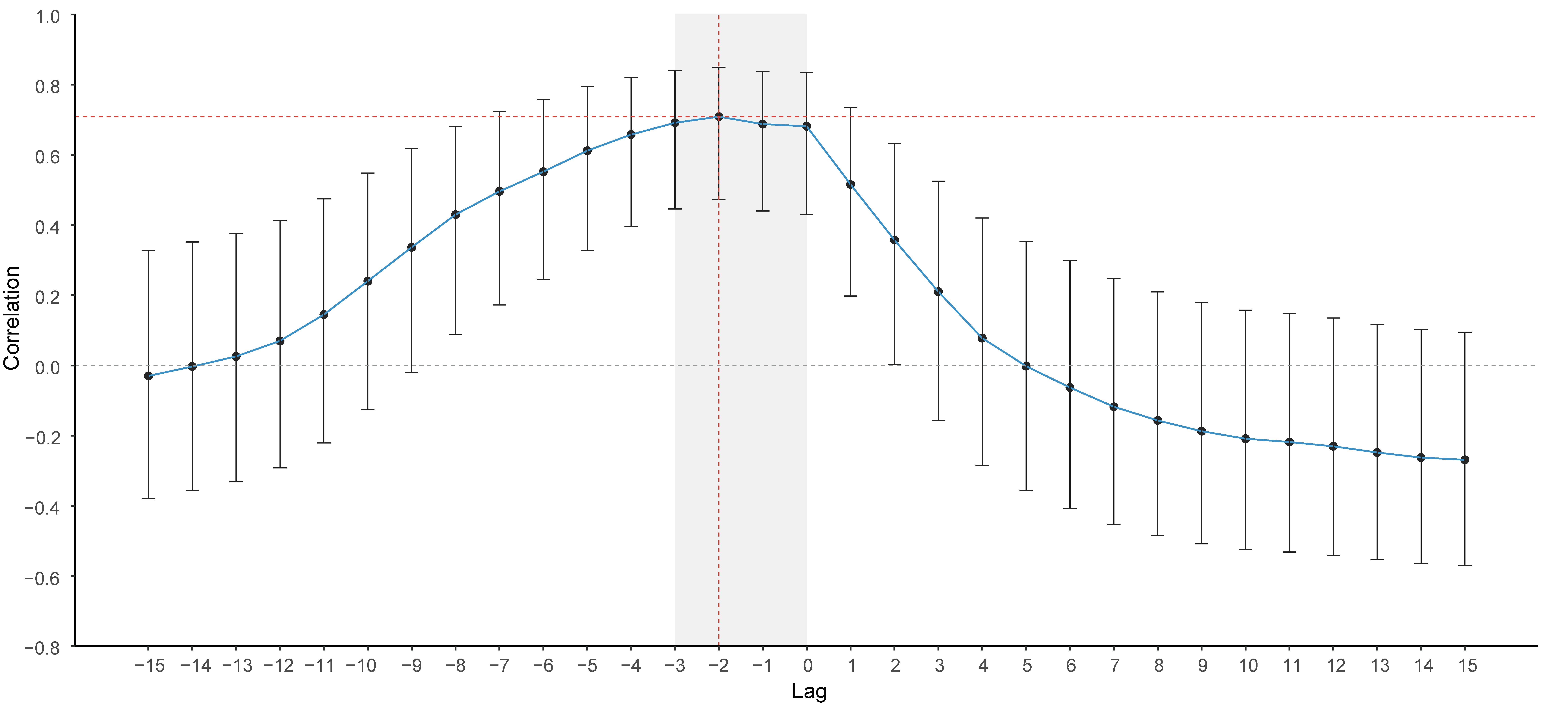
**

**Multimedia Appendix 3.** Correlations between mobility volume and growth rate ratio at different time lags (in days).

An optimal lag of 2 days is noted by the vertical red dotted line.
