# Supplementary material for: Impact of Human Mobility on COVID-19 Transmission According to Mobility Distance, Location, and Demographic Factors in the Greater Bay Area of China: Population-Based Study
Source: JMIR Public Health Surveill. 2023 Apr 26;9:e39588. doi: 10.2196/39588 (PMC10138924; doi:10.2196/39588)
Supplement: Multimedia Appendix 4 [file publichealth_v9i1e39588_app4.doc]

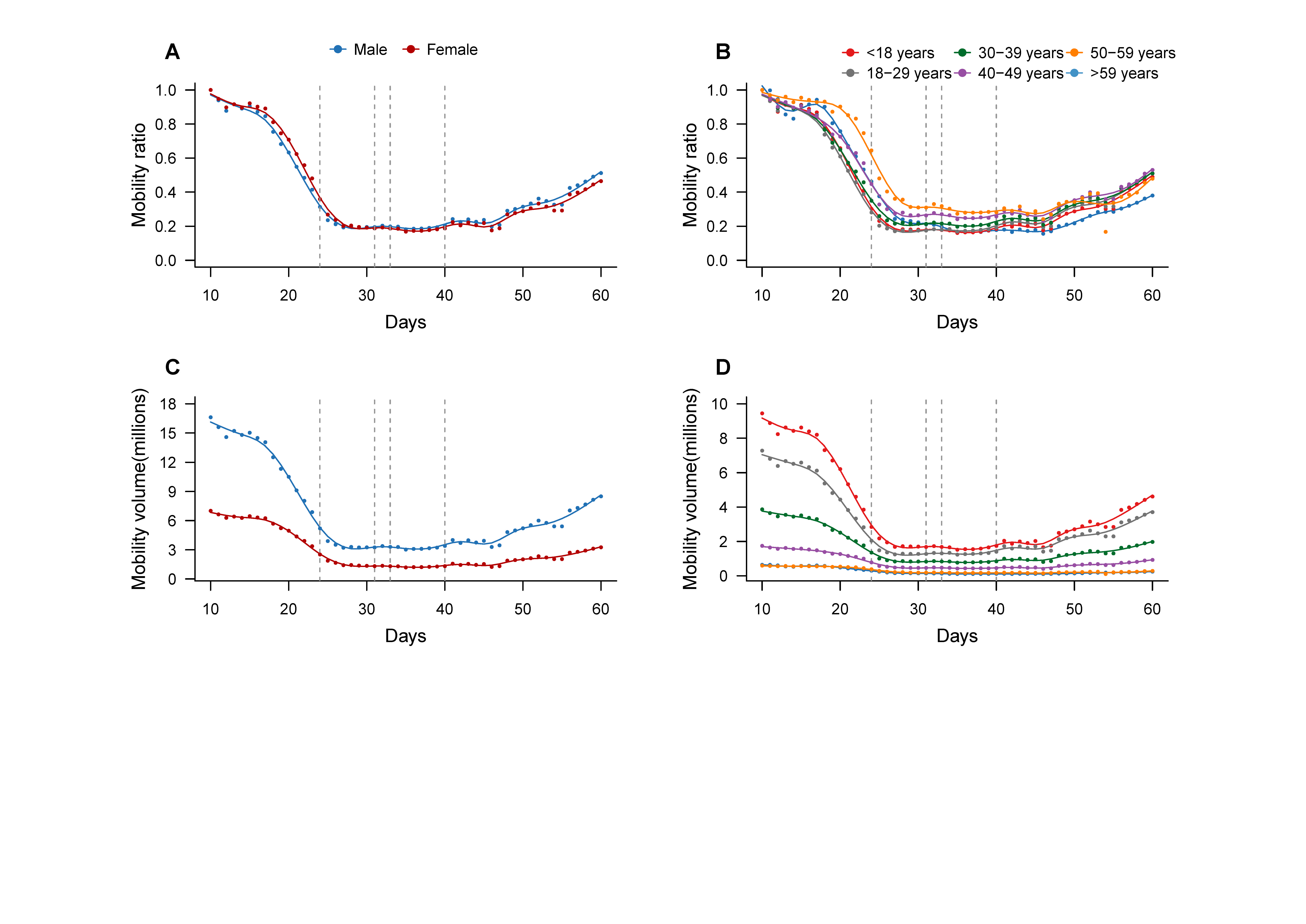


**Multimedia Appendix 4.** The magnitude of mobility volume change for various demographic groups.

**(A)** The mobility ratio compared with the normal days of 2020 for male and female and **(B)** various age groups**. (C)**The raw data of mobility volume for male and female, and **(D)** age groups. The dots represent the observed data and the plotted lines are smoothed by a generalized additive model. “Days” represents the days since Jan 1^st^, 2020. Dashed lines represent the main public health interventions.
