# Supplementary material for: Impact of Human Mobility on COVID-19 Transmission According to Mobility Distance, Location, and Demographic Factors in the Greater Bay Area of China: Population-Based Study
Source: JMIR Public Health Surveill. 2023 Apr 26;9:e39588. doi: 10.2196/39588 (PMC10138924; doi:10.2196/39588)
Supplement: Multimedia Appendix 5 [file publichealth_v9i1e39588_app5.doc]

**
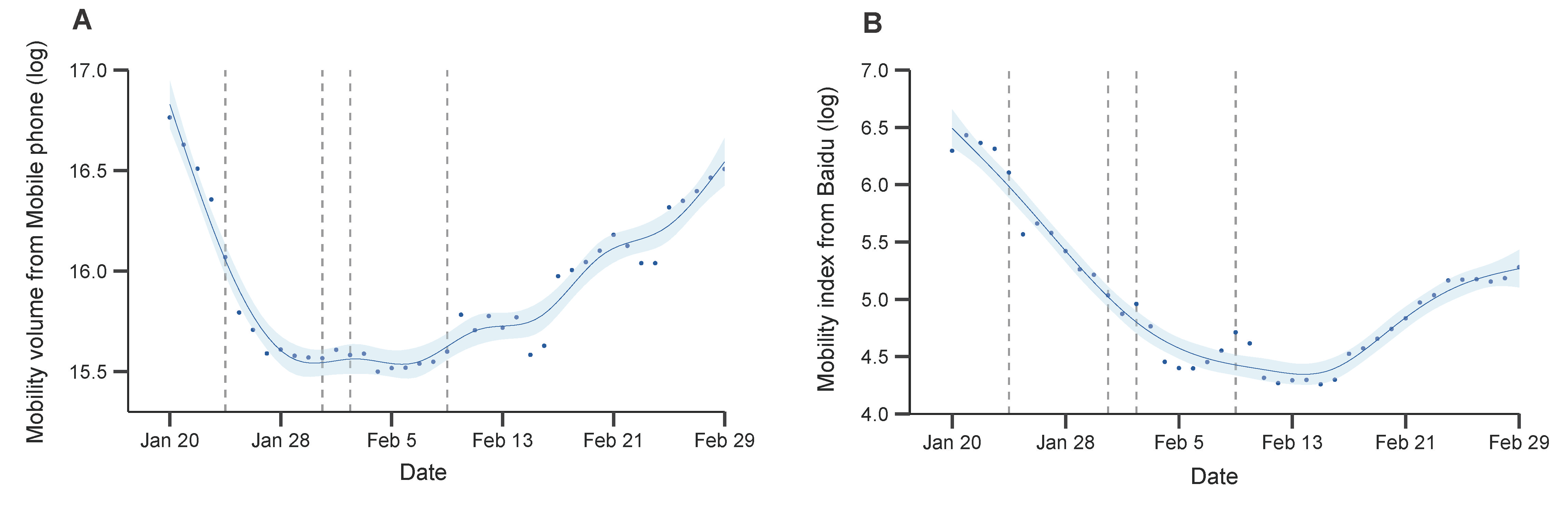
**

**Multimedia Appendix 5.** The time series of mobility volume from mobile phone data in the Greater Bay Area, China and mobility index from Baidu for the whole country of China.

The dots represent the raw data (log) and the plotted lines are smoothed by a generalized additive model with 95% CI. Dashed lines represent the main public health interventions.
